# A Survey on Using Kolmogorov Complexity in Cybersecurity

**DOI:** 10.3390/e21121196

**Published:** 2019-12-05

**Authors:** João S. Resende, Rolando Martins, Luís Antunes

**Affiliations:** Computer Science Department, Faculty of Science, University of Porto, Rua do Campo Alegre 1021/1055, 4169-007 Porto, Portugal; rmartins@dcc.fc.up.pt (R.M.); lfa@fc.up.pt (L.A.)

**Keywords:** NCD, CDM, LZJD, Kolmogorov, security, privacy, cybersecurity

## Abstract

Security and privacy concerns are challenging the way users interact with devices. The number of devices connected to a home or enterprise network increases every day. Nowadays, the security of information systems is relevant as user information is constantly being shared and moving in the cloud; however, there are still many problems such as, unsecured web interfaces, weak authentication, insecure networks, lack of encryption, among others, that make services insecure. The software implementations that are currently deployed in companies should have updates and control, as cybersecurity threats increasingly appearing over time. There is already some research towards solutions and methods to predict new attacks or classify variants of previous known attacks, such as (algorithmic) information theory. This survey combines all relevant applications of this topic (also known as Kolmogorov Complexity) in the security and privacy domains. The use of Kolmogorov-based approaches is resource-focused without the need for specific knowledge of the topic under analysis. We have defined a taxonomy with already existing work to classify their different application areas and open up new research questions.

## 1. Introduction

Cybersecurity experts develop mechanism to protect users and devices, but there is a constant “arms race” between attackers and defenders. Security and research teams work every day on solutions to audit and mitigate attacks. The number of challenges ranges from zero-day detection, software vulnerabilities with known prior attacks or social engineering attacks.

These challenges have led to the production of many security and privacy protocols, and there are security specialists in companies where their job is to analyze implementations and enhance security mechanisms to prevent attackers from exploiting potential flaws and/or vulnerabilities.

However, from a security expert point of view, it is difficult to learn each protocol/implementation to analyze the security to be implemented in the infrastructure.

To analyze the security of implementations, many companies prefer open source protocols because it allows them to make a more informed choice about the security of a software [1]. Despite of having benefits of using open source software, it does not provide security requirements by itself, because there is a need to have continuous auditing of the code.

A security auditor would benefit from a solution that would check for new iterations without needing to understand the entire implementation in detail.

One possible solution is to use Kolmogorov Complexity approach, typically feature free and not requiring knowledge of the protocol itself to produce good results. This paper describes Kolmogorov Complexity methods, such as Normalized Compression Distance (NCD), Compression-based Dissimilarity Measure (CDM), or Lempel–Ziv Jaccard Distance (LZJD), as well as multiple application scenarios of each method in different cybersecurity settings.

For the categorization of the application scenarios, we propose a well-defined taxonomy. This categorization allows the description of the state of the art with a precise sense of the context of what has been done and what future contributions Kolmogorov Complexity can make.

The challenges for cybersecurity experts are considerable, including Privacy, Next-Generation Secure Internet, Trusted Systems, Identity Management and Global Scale Techniques, and Usable Security [2]. Kolmogorov Complexity and the NCD can impact on all of these categories, particularly in the process of validation and automatization of features to avoid human error and improve code quality and security towards trustworthy systems.

This work is inspired by the authors of [3], who provide research on machine learning and deep learning methods for cybersecurity, focusing on summarizing previous research and comparing it. The authors also focus on what information previous works use to produce their results (datasets and models).

This paper is designed to provide an easy entry point for non-specialists and also to benefit experienced researchers following state-of-the-art applications of Kolmogorov’s complexity related measures to information security and privacy.

In addition to show its application scenarios and identifying solutions for each scenario, we also open new research questions.

This work impacts on the recognition of Kolmogorov’s complexity as a way to prevent and detect cyber threats, guiding research on new and innovative approaches on this field.

In brief, this paper wants to answer the following Research Questions (RQ) regarding the Kolmogorov Complexity.

RQ1: What are the domains where it can impact?
RQ2: Can it be an efficient solution to meet the Cybersecurity requirements?
RQ3: Can its approximations be applied in new domains?

The paper is divided into the following sections. Section 1 is the introduction; Section 2 discusses Kolmogorov complexity notions and practical formulas; Section 3 describes Applications Scenarios with a novel taxonomy allowing classification of solutions in groups; Section 4 has a discussion of the findings, with an attempt to explore future research on privacy and security challenges along with directions for Kolmogorov Complexity improve the usability; and Section 5 shows the conclusions according to the Cybersecurity scenarios.

## 2. Kolmogorov Complexity

Kolmogorov complexity [4], or algorithmic information theory, is a mathematically sound theory measuring the amount of information in an individual object as its smallest representation. This measure is noncomputable; recently, its approximation, based on compression, has been used in computer science and a plethora of other scientific disciplines.

Normalized Information Distance (NID), based on Kolmogorov Complexity, measures the minimal amount of information required to translate between two objects. It is well known that this measure is also noncomputable, nevertheless we can approximate it by using standard compressors. In the scope of statistical or clustering methods, it is important to measure the absolute information distance between individual objects.

There are a number of different implementations trying to approximate the NID, the most known implementation is the Normalized Compresion Distance (NCD).

### 2.1. The Normalized Compression Distance

Cilibrasi and Vitány [5] introduced a new clustering method based on the NID. This method is powerful; it does not need any background knowledge of a specific area to extract patterns independent of the domain, allowing to cluster heterogeneous data and anomaly detection in time sequences. Some applications are in music [6] or heart rate anomaly detection [7].

This method uses compressors to reduce a file to the small representation and uses file size to perform mathematical calculation that cause similar files to produce similar results. The NCD was first proposed by Ming Li et al. [8], as a real-world approximation to the notion of NID.

Some experiments shown by Rudi Cilibrasi et al. [5], regarding the impact of NCD on clustering, show that the NCD is a (quasi-)universal similarity metric to a normal reference compressor. To apply NCD we need to choose a compressor to make an approximation of the smallest representation of the program.

(1) $$NCD\left(x,y\right)=\frac{C(xy)-min\left(C(x),C(y)\right)}{max\left(C(x),C(y)\right)}$$

In the Formula (1), we give as input two different files: *x* and *y*; the result of C(xy) represents the file size resulting from compressing concatenation of *x* with *y*. *C(x)* and *C(y)* are the compression size of *x* and *y*, respectively.

NCD function provides values in the range 0≤r≤1+e representing the difference of two files. Results of NCD closer to zero represent more similar objects (files); results closer to one are more distinguishable objects. The *e* is due to imperfections in the compression algorithms, but for most standard types of compression [8] is unlikely to see an *e* above 0.1. Tests with *PMZ* show that values of NCD above 1 are not normal.

The NCD matrix is a matrix where the comparison of *n* samples is performed in a n×n matrix, resulting in each object (file) being compared to each other and to itself. This matrix can be used in data mining techniques such as clustering, where each entry can be classified accordingly to similar objects.

#### 2.1.1. Optimizations of NCD

There are two types of NCD optimizations: Interleaving and NCD-shuffle proposed by Rebecca Borbely et al. [9]. These solutions arise from the varying performance on large files (depending on the compressor).

When NCD performs the computations of C(xy), the goal is to compress the values of both files to help to determine the similarity, but compressors have some limitations. Algorithms like bz2 and zlib have an explicit block size as a limiting factor, and lzma has a finite dictionary size. This dictionary has repeated sentences representations translated into smaller symbols, allowing to compress original files and their dictionary into new compressed files. As it processes its input, the dictionary grows. When a dictionary size exceeds, the algorithm starts with an empty dictionary and if this occurs without processing the entire *x* from C(xy), there is not a benefit of compress *x* followed of *y*. This makes the compression obtain similar values of C(xy) to C(x)+C(y).

Interleaving is a solution that attempts to calculate the NCD at a specific size(appropriated for the compression algorithm), assuming that *x* and *y* have the common parts aligned. Unlike Interleaving, NCD-shuffle splits files into parts of a specific size (appropriated for the compression algorithm), calculates the most similar block of *x* present in *y*, and aligns them to take advantage of compression capability.

### 2.2. Compression-Based Dissimilarity Measure

CDM proposed by Eamonn Keogh et al. [10] is inspired by bioinformatics, learning, and computational theory, and has been applied by different authors in other domains, such as data linkage and reduplication problems [11].

(2) $$CDM\left(x,y\right)=\frac{C(xy)}{C(x)+C(y)}$$

The formula to compute the CDM is represented in the Equation (2). The input are two different files *x* and *y*. The result of C(xy) represents the size of the file resultant from the compression of the concatenation of *x* with *y* and C(x) and C(y) is the results of the file compressed. Such as in NCD, values close to 1 occur when *x* and *y* are not related, and values close to 0 occur when *x* is very similar to *y*. The values of CDM varies by the compressor used.

### 2.3. Lempel–Ziv Jaccard Distance

LZJD [12] is a new derivation of compressor use to represent the NID and is inspired by the NCD. This measure, instead of taking advantages of the object’s compression size, uses the Lempel–Ziv (LZ) technique to create a compression dictionary of previously seen subsequences; therefore, in this scenario, the compression iteration is done only because it is required to generate the dictionary. The LZSet method is used to convert a sequence of bytes into a set of byte subsequences.

(3) LZJD(x,y)=1−J(LZSet(x),LZSet(y))

The LZJD is defined by Equation (3). The *LZSet* represents the compression dictionary and *J* is the Jacard similarity is the cardinality of the intersection of two sets divided by the cardinality of their union Equation (4).

(4) $$J\left(A,B\right)=\frac{\left|A\cap B\right|}{A\cup B}$$

### 2.4. Normalized Relative Compression

Normalized Relative Compression(NRC) [13] is another approximation to NID, but compared to other approaches, is the C(x||y), which represents the compression of *x* relative to *y*.

Formula (5) represents the practical implementation of this approach. The inputs are two different files: *x* and *y* and the |x| is the length of *x*. The value of C(x||y) varies depending on the object, if *x* can be constructed from *y* the value is 0, if *x* can not be constructed from *y* the value is the size of *x*.

(5) $$NRC\left(x||y\right)=\frac{C(x||y)}{\left|x\right|}$$

## 3. Kolmogorov Complexity Application Scenarios

Kolmogorov complexity-based approaches can be applied to various scenarios. This section describes the different application domains organized into a taxonomy. To build this, we started by surveying publications that use NCD, LZJD, CDM and NRC on information security topics to understand its impact. We then use a selection methodology for the different domains, based on previous work in the different areas. In addition, we have included a review of previous work and future research directions for each domain.

**Figure 1:**
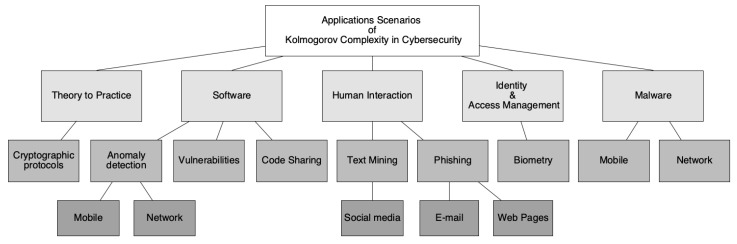
Taxonomy.

Figure 1 presents the taxonomy that is organized in five categories:

– Human (user) Interaction: Today’s systems need human-in-the-loop interactions. Users are very susceptible to errors and this leads to challenges that can be solved [14,15,16,17,18,19,20,21,22,23,24].
– Software: The rapid pace of how the software is being used makes it almost impossible not to use open forums/repositories to solve our needs. This creates software security breaches that compromise user data even on data in transit [25,26,27,28,29,30,31,32].
– Malware: In recent years, companies have been attacked with malware. The scalability to mount an attack on multiple institutions at once is not expensive compared to the reward. This leads to blackmail to companies/users, disclosure of files or credit card data in the web, or encryption of databases and files [9,12,33,34,35,36,37].
– Identity/Authentication: With the exponential growth of wearables devices, users and devices will need to manage a new authentication mechanism to pair and interact with enhanced features, gathering information from the ECG (electrocardiogram) or similar, for example [38,39,40,41,42].
– Theory to practice: There are some protocols that enforce privacy and security in communications between users. With the emergence of new cyberattacks and with the recent developments in academia regarding privacy policies imposed by rules to protect user privacy, such as GDPR, it is of utmost important to validate existing implementations to ensure user privacy [43].

The following subsections introduce each of the topics represented in the Figure 1.

### 3.1. Human Interactions

Corporations have multiple layers composed by network and physical security. Network security has different components, such as firewalls, antivirus, or policies. However, physical security consists of barriers to prevent people from entering the institution, for example. In this scenario, many authors claim that the weakest part of the systems in the security chain is the human [44].

There are many solutions to mitigate this type of attacks, but there is no effective solution to solve the problem because employees can be triggered by any means to do something wrong.

Kolmogorov Complexity has been used for early detection of fake content. Two application scenarios are Phishing and Text Mining.

#### 3.1.1. Phishing

Phishing is an activity by one attacker to impersonate another. This type of phishing attack focuses mainly on two versions: e-mail and web pages.

On web pages, for example, we can see the similarity between an old and a new page to understand the page’s evolution according to a threshold. This allows to dissuade the victim from clicking or interacting with a web page similar to the one that the victim usually uses with an identical link and content. This way, it tries to get the user to log in to this page for more sensitive information and credentials, such as credit/debit card details, for example [45].

In emails, a person or entity may be compromised by an email sent from an illegitimate source, pretending to impersonate other and requesting personal information (for example, a CEO requesting the transfer/raise of an employee). Kathryn Parsons et al. [46] study the ability to classify an email as phishing or not, and the conclusions are that 42% of the email was misclassified. The practical solution to this is to develop new and innovative approaches to automatically detect SPAM/HAM.

##### E-mail

E-mail can be classified between SPAM, HAM, and legitimate. SPAM is often understood as an electronic system that sends unsolicited bulk messages to random locations or specific destinations, such as the “Nigerian letter” [47]. HAM is a type of email sent from a mailing list subscribed by an user directly or indirectly. For example, after submitting an article, users automatically subscribe to a conference mailing list that sent all new articles published and call for papers. The classification of incoming e-mail is an open issue in security research. With the growth of machine learning technologies, attackers gain new ways to explore and exploit new types of attack vectors that customize SPAM e-mails to targeted users. NCD has contributed to the detection of these type of attacks [14,15,16].

Delany and Bridge [16] focus on feature-free distance measure and compare them. In this paper, the authors study NCD and CDM and introduced the concept of drift that represents a dynamic target concept. The target concept can be viewed as a specific list of e-mails that changes over time according to world events or seasons. The changes are also affected by people’s interests, for example, conference or seminar reminders can become unpleasant. In brief, a subset of e-mails is selected according to the time of the year or the more recent e-mails of each user. The advantages of using the concept drift influence performance because a short list of emails is used to compare and update, allowing detection of new type of e-mails in use without processing all historical e-mail (an example is e-mail with historical facts that loose value over time).

For the study, the authors use a private dataset containing e-mails received by a set of users for one year. Comparison of NCD with CDM shows that NCD outperforms CDM and the concept drift decreases misclassification. Also, the results show that the accuracy of NCD compared with Feature-Based Distance Measure (FDM) is better or equal. An advantage of use NCD is the cost to setup and simpler periodic maintenance demands.

Other work on SPAM detection uses the Text REtrieval Conference (TREC) dataset, organized in the year 2005. The dataset [48] contains 48,360 SPAM emails and 36,450 HAM emails. The authors Prilepok et al. [14] performed a test with different compressors and concluded that, using the NCD, the HAM classification was independent of the compressor. However, SPAM was compressor dependent, but ranged from 66% with Burrows Wheeler with Adaptive Huffman Encoding, RLE and Fibonacci Encoding up to 90% Adaptive Huffman. A more recent approach to Prilepock et al. [15] included the previous TREC dataset with SpamAssassin Public Corpus and confirmed the effectiveness of this approach. Also, authors perform a 2% improvement if the spam filter uses signatures. Signatures contain information extracted from e-mails, but are much smaller and lead to a quick response, because it is easier to verify that a signature is present in a predefined list than to compare incoming e-mail with a SPAM and a HAM dataset by similarity (signatures only work if the message is exactly the same).

##### Web Pages

Phishing web pages have traditionally been detect by DOM tree, HTML code or link structure of a page, but there are also new approaches to take advantage of Supersignals [49] and Gestalt [50]. T.-C. Chen et al. [17] introduced a system that takes advantage of these concepts to analyse the Web page as indivisible entities (i.e., a whole) to be classified based in the human perceptions (visual representation). To implement and compare the results of NCD in the detection of phishing web sites, the authors used PhishTank [51]. PhishTank is an open platform that allow users to query threads and report a website as phishing or not to other users. The results based on a series of experiments demonstrate that this approach is capable of classify and cluster between similar and dissimilar web pages. As future work, the authors propose a creation of an antiphishing system. This system was then introduced by T.-C. Chen et al. [18]. To accomplish a better performance, the authors also implement the possibility of adding a blacklist to the antiphishing platform allowing owners to submit a sample of a fraudulent web page.

Alberto Bartoli et al. [19] explore the feasibility of NCD as a phishing mechanism in web pages, based on a real environment. The authors propose the possibility of an attacker learn the NCD formula and then be capable of compute a page P that is similar to the targeted page P’, ensuring that NCD(p,p’) is a higher value to mislead the behavior of the phishing algorithm. The authors also explore what can be a significant difference, and the results include changes that only slightly change the appearance, the brightness of the background color or the zoom of the entire website will not be noticed by the user contrarily to the opposite value of NCD. They conclude that is complex to detect SPAM only with NCD, but further iterations must be done to understand relevant parts of the web page (i.e., focus only in the subset of the webpage that contains the login mechanism).

#### 3.1.2. Text Analytic

Recent developments in text analytic focus in discovery of patterns specially in long text. In a recent work [52], the authors suggest that we can use these to detect plagiarism in e-Learning/e-Publishing systems, assist in preparation of expert reports in criminology, the identification of channels of threats in cybersecurity, and providing digital libraries with tools for studying writing style. The authors also compare the results of compression based algorithms with n-grams and conclude that the precision reaches 20%. However, NCD, as a file fragment classifier, can bridge and create mechanism to detect the authorship, example of this will be source code replication or text available on the web can be copy by a individual with miner changes to mislead users, an example of this is change small parts of the text. The work in this field is extensive and show the variety of scenarios where NCD can be applied in long text analytic [53,54,55,56,57,58].

##### Social Media

With the growth of the number of Internet users and the importance, security agencies need to gather and collect information to understand user behaviors. There are two major goals: detect fakenews and detect physical or cyberattacks. Fakenews is one of the most relevant problems for security researches, there are many research challenges on the detection and reduction in the spread of fakenews [59]; also, there is a topics and threads that allow authorities to detect events and trending topics and eventually illegal events [60,61,62,63] or to detect patterns in post from social media in order to understand and detect users that control multiple social media accounts.

The work with NCD in this domains focus initially in the detection of suspicious accounts; Alami and Beqqali [20] introduced a method using suspicious terms collected in a data base (manually added), allowing classification of the entire message. This is done by dividing and classifying each word in two categories Normal or Suspicious. The results were developed using a Twitter repository of messages from 2012 [64]. An extension of this work by Alami and Beqqali [21] was proposed, the motivation focus in the need of analyzing and create mechanism to disambiguation, in this scenario they classify the hashtag, this allow to identify hashtag and message that should be threated as security problems, and can have a specific dictionary, contrarily to have a fix dataset that took more time to compute. The practical implementations of this in a real scenario was done by Rasheed [22], where it has also used dataset with sentiment score to improve the dataset search.

Regarding the detection of multiple accounts, a recent article used NCD for this detection [23,24] created two datasets containing information from Twitter feeds from individuals who each control multiple Twitter accounts and one that merged with the StackExchange, most of the techniques used failed when detecting same user accounts, as suggested by the authors temporal and semantic approaches failed because users split accounts for example by topics meaning that the words are different, also the publication ratio can also be different, meaning that NCD has the best in the first dataset, by contrast, in the second it has not the best, the authors suggest that based on the difference between the text sizes the NCD could not overcome this issue and did not results well.

### 3.2. Software

With the exponential growth of smart devices and the proliferation of laptops and cloud computing, there is a need for autonomous tools that together can work and reinforce practices to contribute to better understanding and solving challenges such as finding vulnerability across multiple layers of code/projects or understanding unsafe programming practices.

#### 3.2.1. Code Sharing and Vulnerabilities

Software development has been empowered by sharing/colaborative platforms. An example of this sharing platforms are Github and Stack Overflow. Github allows users to collaborate in projects widely available, and stack overflow is a discussion forum that allows users to expose questions or to answer another users about software-related problems. However, this platform also has issues such as non-expert answers or the efficiency/safety of solutions that are not considered either. These pieces of code can often introduce vulnerabilities in real-world deployments [65]. These platforms are immediately recommended by search engines when there are code issues searched by developers, and often they just copy/paste the code to solve a problem without auditing [66,67,68].

When vulnerabilities are found, they must be searched along all code from an enterprise to fix the issue. There are many commercial applications that allow a developer to find security vulnerabilities on source code. The approximations was first introduced by Takashi Ishio [25], where NCD is used after a user finds a vulnerability. This process is simple: it only requires searching similar patterns in the other files of the project to patch the same error. The tool is compared to a grep style detection but with the benefits of NCD. Grep command only finds the exact type of code searched but with NCD, it is possible to search for (very) similar changes, for example, the introduction of a variable or the change of a loop variable from “i” to ‘j”. The NCD will be capable of detect these type of changes, by acting dynamically, but the grep command only search for the pattern ignoring all variations.

The BinAuthor [26] is a framework for identifying the authors of program binaries and it uses NCD as a way of identify structural similarity. The results indicate that the precision of these techniques depends on the number of authors, for example approximately 50 authors or more make the accuracy drops to 45%. The difference between authors with advances skills can be easily identified compared to authors who do not have much experience.

#### 3.2.2. Anomaly Detection

With the proliferation of network/mobile usage, many companies move the services to cloud, such as banks, allowing customers to check balances or transfer funds between accounts [69]. This challenges the way security experts look for security holes because hacking can be from different sources, such as network connections, or by simply modifying the original application to perform different requests.

##### Network

When software is deployed on a large scale, companies need to infer what is happening with software to prevent cyber crime. It is important to debug for potential errors or to audit the system. Typically, the first solution is to store all log information in empty files for postprocessing; this information can be, for example, the number of attempts to log on to a system, the source IP, the destination IP (in the case of central logs), and the ports used. The possibilities of using this information have been explored by many authors with proven results [70]. A solution proposed by Gonzalo de la Torre-Abaitua et al. [27,28], shows the possibility of using NCD to perform proper analysis of the log files of multiple services to detect anomalies at an early stage. The authors identify five main topics, but the two most important are URL identification domain generation algorithms and Domain Generation Algorithms (DGA), which are important when parsing log files. DGA are algorithms and DNS names that are periodically generate a domain name that try to pass by a real domain name. Malicious URL is normally a request made by users or machine for a web service that may follow a bad parameter specification to try to enter in a debug mode or administration console, for example. In this scenario, the authors used a public CSIC dataset [71] containing both safe and unsafe HTTP queries, and they compare the results with similar approaches of anomaly detection, and the results are very similar. The advantages of using NCD, is that there is no need for feature selection, depending only on the compression algorithm. DGA solutions have the ability to detect fake domains on the network, for example, when software is vulnerable and begins to connect to remote sites and exchange information. In this scenario, NCD calculates similarity and produces better results with detection known systems, but using a small percentage of domains to train the model.

Although our approach does not always improve the best results obtained by other researches based on the same data, it leads to a similar performance avoiding the computational cost of any previous feature selection process.

Christina Ting et al. [29] adopted the use of NCD in the analysis of DNS queries. The authors used an Intrusion detection evaluation dataset (ISCXIDS2012) [72] from the Canadian Institute for Cybersecurity where they extracted the DNS queries and answers. The authors introduce the slice compression mechanism that is responsible for removing the applications data portions letting only NCD with the protocol differences (i.e., the TCP connections loses all the application data letting only the information of the headers available for processing). The results show that this can be a good approach to apply to network protocols.

##### Mobile

Static analytic of Android application is used in scenarios such as testing if obfuscation algorithms are working properly, testing if android applications are similar to the previous version, and if the code does not include bad code, such as blockchain mining functionality or malware distribution [73]. The first proposal by Anthony Desnos [30] tries to calculate similarity after decompiling the application and search based on an algorithm that generates the signatures of each method, identify unchanged methods, and search by similar methods using NCD. The authors make a test for the version of Skype where 165 changes were detected by NCD. This changes can then be classified by degree of importance. A recent work from Sreesh Kishore et al. [31] proposes a system that uses NCD to compute the app lineage or detect malicious components in applications, because they use birthmarks from 60 APK. Birthmarks are intrinsic characteristics of an application that can be uniquely identified. In java, the proposed birthmarks are Used Classes, Constant Values in Field Variables, Sequence of Method Calls, and Inheritance Structure proposed by Tamada et al. [32]. The system proposed to collect applications from multiple sources and split them between test and baseline applications. Test applications are crawled from the web, such as open repository or none standard markets and the baseline applications are collected from the Google Play. Birthmarks are then extracted from applications and sent to the compute engine, where if the applications have a NCD of less then 0.4, they are considered similar; otherwise, they are marked as fake and not safe. They compare the results with *androsim* and this system improved state-of-the-art detection of similar classes with 100% precision compared with 45.5%.

### 3.3. Malware Classification

As mentioned, phishing allows an attacker to spread and impersonate companies. This type of impersonation attack can cause a user to download files that contain malware attacks. Therefore, in addition to solve Phishing problems, it is also necessary to solve issues related with malware attacks. Malware includes different types of attacks, such as viruses, worms and spyware. The malware industry is a well-organized and well-funded marketplace dedicated to bypassing traditional enterprise security systems. Once a computer is compromised, the entire infrastructure is in a critical state. The impact can be huge on the brand or affect the business model, exposing personal or customer information in many ways [74,75]. To help predict and improve the malware rating that Microsoft released in 2015 as a challenge, the goal was to become effective at analyzing and classifying large amounts of files. These files should be grouped and identify their families. Traditional solutions address malware detection by running and searching predefined bit patterns (signatures), which were previously classified as malware in the virus database. The functionality of this is essentially limited to previously known attacks, which means that the changes performed, will completely change the malware signature, making it new and undetected malware, called metamorphic or polymorphic malware. NCD can especially help with these small changes by calculating object similarity, making the system more robust to character-level adversarial attacks, such as polymorphic/metamorphic viruses.

Metamorphic malware uses code obfuscation techniques to change its internal code structure while maintaining its malicious functionality during each propagation. To improve and solve this problem using NCD, Michael Bailey et al. [34] introduced the use of NCD, but instead of applying to malware code or executable, it uses behavior. Malware behavior has already been proposed as a solution to deal with polymorphism and metamorphism, where malware changes its visible sequence of instructions as it spreads. To do this, authors use user-visible system state (such as open files, created processes), and then use it as the malware fingerprint. These fingerprints are more stable than abstract or dead code; they then run all malware in a controlled environment and collect this information. This allows authors to compare and identify the type of malware exposed in each situation by those that reflect similar classes of behavior.

Binary malware analysis is classified as static analysis where code is not executed; the advantages of this type of analysis are the security of not having to create a safe environment. There are two different works with compression in this kind of scenario ([9,35]). Rebecca Schuller Borbely [9], study the definition of normal compressor to understand the best compressor for Microsoft challenge analysis [76]. The authors introduce two changes to the NCD formula to improve the compression functionality using Interleaving and NCD-shuffle (see Section 2.1.1). These changes produce a performance increase, for example, *zlib* increased from 50.5% to 83.9% in malware classification. Another work from Alshahwan et al. [35] inspired by Wehner’s work [77] attempts to classify malware based on executable binaries. The results show that a malware reported within a short period of time (i.e., a few days) is more homogeneous than malware reported over a longer period of time (i.e., a year). The authors carry out several studies and conclude that, by the time of 2015, the detection by the platforms present on VirusTotal site was worse in all cases. A more recent study also outperformed the VirusTotal in 2019 [78].

The LZJD [12,36] helps in the malware classification and introduced a comparison with NCD. The results were interesting not only based on the improvement results, but on Microsoft’s dataset, specially in the cost of computation, which makes the computation faster and simpler. The authors show the results of Microsoft dataset analyzing with NCD in only 10% of the data and KNN with k = 1, and this increase predictions from 58.1% accuracy with NCD to 98.2% with LZJD. The results show that this is a good result for malware classification. The same results were tested with the Drebin dataset [79], which contains APKs from different Android applications, and from 67.2% with NCD, the results with LZJD raised to 81.4%. To improve the system the authors also propose a stochastic component to enhance the behaviour of the compressing algorithm [33].

There are other approaches that focus on using compression Approximate Minimum Description Length (AMDL) and Best-Compression Neighbor(BCN) [80,81], but this is not derived from Kolmogorov Complexity.

There is some work that focuses on comparing NCD with other market solutions. A recent paper by Houtan Faridi et al. [37] concludes that NCD with a similarity threshold of 0.4, compared with other approaches, is the best metric for detecting malware.

Table 1 represents and overview these articles. It focus on the format of malware used, the type of Kolmogorov formula used, and the dataset used to test the solutions. We show in the table how to compare different approaches with the same dataset, otherwise we cannot choose the best malware classification solutions. There is, as shown in the previous sections, a greater impact of NCD compared to LZJD.

For future work, after performing a comparison with a fixed dataset of all the approaches, it is also important to test the difference in a dataset of using traces compared with Binary or hexadecimal.

### 3.4. Identity & Authentication

Traditional mobile authentication/identity, such as passwords or fingerprint techniques, are vulnerable to attacks [82]. One of the new mechanisms to solve the problem of authentication, is the usage of the smart lock technology. A smartwatch can be configured with the smartphone in order to unlock by closeness. This way, smartwatch can identify a user [83]. These types of solutions are being proposed, such as the use of Electrocardiogram (ECG), to authenticate/identify a specific person. ECG is the representation of the electrical signal that comes from the contraction of the heart muscles, indirectly it represents the flow of blood inside the heart [39]. A study to understand the number of heartbeats needed to uniquely identify a person using NRC, shows that it is possible with only 5 to 12 heartbeats, maintaining the accuracy between 75 and 80% [38].

Arteaga-Falconi et al. [40] introduced ECG for Mobile devices to work as an authentication system. The benefits of this type of systems for authentication are important for ensuring the presence of the user without possibility of cloning the identity.

At this moment, the use of NCD in the authentication of mobile devices or in other systems is an open challenge, but there are already some approaches in the identification of a user based in ECG and compression mechanisms.

Past works have been focus on the identity based on known datasets that try to identify users. Carvalho et al. [41] introduced NRC with an improvement compared to past works with the same dataset. Later, Bras et al. [42] improved the results from 80% to near 90% in identification of users.

### 3.5. Theory to Practice

There are protocols that enforce privacy and security in communications between users. With recent developments in academia regarding privacy policies imposed by rules to protect user privacy, such as GDPR, it is essential to validate existing implementations to ensure user privacy.

#### Cryptographic Protocols

There is always a difference between theoretical and the equivalent practical implementations. The theoretical approaches of cryptographic protocols, for example, are always published mathematical proofs. However, there are a lot of open source implementations of these theoretical protocols, that are used by companies in production, and there is no validation of the implementation regarding all the mathematical proofs of the theory.

The problem of Software Engineering Practice in Scientific Programming has already been introduce by Tim Storer [84], where it surveys facts from this problem that arise from the late 1960s with work from Naylor and Finger [85].

NCD was introduced as a novel approach to solve these problems. An example of this is the implementation analysis of a cryptographic protocol (Multiparty Computation (MPC)), where Kolmogorov Complexity helps to detect anomaly patterns in the properties that are guaranteed by the theory of the protocol [43].

MPC was formally introduced as secure two-party computation in 1982 [86,87,88]. Andrew Yao introduced the millionaires’ problem in 1982, the seminal secure multiparty computation example/problem. The scenario consists of two parties whom are both interested in knowing which of them is richer without revealing their inputs (i.e., their actual wealth). In this scenario, each party uses respective inputs *x* and *y* denoting their salaries. The goal is to find the highest salary, without revealing their respective salaries. Mathematically, this can be achieved by computing

f(x,y)=max(x,y)

At the end of the protocol, each participant will get only the result of the function *f*, without getting anything else about the other party’s input, i.e., the secret inputs will not be revealed. This protocol has to ensure two main security properties:

– **Privacy**: The inputs are never revealed to other parties;
– **Correctness**: The output given at the end of the computation is correct.

These security guarantees are to be provided in the presence of adversarial behavior. There are two classic adversary models that are typically considered: semi-honest (where the adversary follows the protocol specification but may try to learn more than allowed from the protocol transcript) and malicious (where the adversary can run any arbitrary polynomial-time attack strategy) [89].

The authors focused in the semi-honest attack, where both users communicate, and passively intercept the information exchanged over MPC. For that, the authors used the network information produced by the implementations to cluster them using NCD. To demonstrate their findings, they created a scenario with the Forbes millionaires. This millionaires were replicated in a laboratory to perform 5 communication between them. With this approach, the results show that only one (ABY [90]) of the four implementations(SPDZ-2 [91], TinyLEGO [92], and DUPLO [93]) is secure.

## 4. Discussion and Future Research Directions

In this section, we aim to discuss the initial defined research questions (see Section 1), suggesting further research directions.

Regarding the question RQ1, the domains can be grouped in five categories: Theory to Practice, Software, Human Interaction, Identity & Access Management, and Malware (see Figure 1).

From all categories, *Human Interaction* is the category with most different sub-domains, because text analytics has been a very active research field over the years, especially regarding the similarity between texts to detect authorship, for example.

Regarding the efficiency of solutions on meeting cybersecurity requirements, there is not a clear answer (RQ2). Kolmogorov Complexity presents academic results that fit the needs from the real world in many domains, but authors of some publication claim some limitations. This limitations mean that in some scenarios, the existing results can be easily misleading. An example of this is the detection of malware for the first time because, if an attacker knows the type of analytic performed, it is possible to compute a new malware solution that is not detected otherwise. By performing random tasks in the execution process, it generates a new type of output of the same attack. BinAuthor [26] is a type of solution that shows multiple applications of Kolmogorov Complexity: it applies it to simple tasks (detection of code structure similarity), and enhances it with external sources of information, such as, in the example of collaborative phishing detection (PhishTank [51]).

So, the answer to the question RQ2 is that it depends on the application scenario.

New scenarios for NCD application to cybersecurity issues are emerging, and this is important for research in the area and possibly to help address efficiency issues. *Identity & Access Management* and *Theory to Practice* domains are the most recent application scenarios to Kolmogorov Complexity, and this is the answer to RQ3. The utility and future work for these specific domains is:

– Identity and Access Management: This field focuses on ECG-based identity validations and authentication. This allows, for example, to collect this information with wearable and process the heartbeat to provide identity, for example, physical access to institutions, login at the computer/mobile phone, or identify patterns about the user feeling when reading an e-mail, among other approximations.
– Theory to Practice: This domain is one of the most important when implementation arise from the theoretical/mathematical proofs to a real world implementations. The first version of the software must always be treated as unsafe and not immediately deployed at scale. The challenges aims for searching patterns in homomorphic encryption, searchable encryption and other similar protocols to measure the security and reliability of the system implementations. These type of protocols can have a high impact on society, but it is important to test and find ways to validate the security and privacy requirements.

### Future Research Directions

Compared to other solutions, deploying Kolmogorov’s complexity needs work to enable the academic community and businesses to begin using it as a true solution to cybersecurity issues. Also taking into account the Figure 1, there are other future research challenges, including those presented during RQ3, in existing domains that focus on framework availability or deployment in the real environment:

– **Software**: In the future, the Software domain needs to be tested in a real environment. In anomaly detection, the challenges is to test with known datasets, such as the summarized by Cinthya Grajeda et al. [94] to understand the usability in new sub-domains. Besides analyzing all the code, there should be a preprocessing of the data, following some rules depending on the implementation and application scenario, to remove external sources of entropy. Regarding Code Sharing and Vulnerabilities, the future directions should focus on the integration into open-source tools to detect events based on multiple sources.
– **Human Interactions**: There is no evaluation in the real world environment of this approach. It is interesting to evaluate phishing detection over time, for example, to see if the solution is viable or not in a real context. Real-world deployments are needed to show the advantages of this approach in cooperation with other tools to test the ability of outperform the state-of-the-art deployments, especially in web pages and e-mail phishing. Further enhancements to Fakenews may benefit to address some open issues proposed in this domain, such as automation and specialized tools [63].
– **Malware**: Regarding Malware, there is a clear need to compare all different articles against a common dataset to compare the performance of each approach. This is especially important when considering Table 1, where all articles are shown to use a different dataset. There are also more datasets available for performance testing [94]. Real application scenarios, in our opinion, should be based on an approach that uses NCD in the tool chain, but uses other external sources of information with known practical results validated by security experts, enhancing these tools.

Future challenges are not only focused on cybersecurity issues, but also on mechanisms to allow a faster pace for Kolmogorov Complexity algorithms. It is necessary to classify and show which formula is the best for classification, using Kolmogorov Complexity, dependent of the application scenario. The result depends on the compression algorithm or the size of the dictionary. All these studies must be done to create mechanisms to clearly understand the functionality and limitations of each one.

The study of the normal compressor definition should be addressed as preliminary theoretical studies have been performed, but no practical analytic is performed. Also, current implementations of the formulas analyzed do not use the same implementations, for example, NCD uses Complearn, which works almost instantly, making NCD one of the most widely used platforms. It is important to create a library that gathers all these different formulas and compressors and show/test what are the best solutions for a specific scenario. LZJD, for example, should be tested in different scenarios as it is one of the most recent approaches.

## 5. Conclusions

Over the years new opportunities and challenges for information theory have emerged. Many research challenges have been focused on the use of Kolmogorov Complexity to compute similarity between objects, especially focused in the medical domains. Current research have become a multidisciplinary subject that includes applications on Machine Learning, for example.

In this article, we review the security and privacy features of the relevant practical implementations of the Kolmogorov Complexity and assess their impact across multiple domains, which is a clear demonstration of the use cases (Figure 1). The use of these technologies in the cybersecurity domain is motivated by the feature free and no parameter to tune. Protocol proliferation contributes to an autonomous framework that can be computed without much required knowledge.

Kolmogorov Complexity focus on the absence of need for in-depth knowledge of the domain data, but this can induce users on trying to use it as a quick fix. However, from our perspective, many times it is necessary to perform preprocessing data such as the authors that analyze the security of MPC [43]. To perform validations, it was necessary to remove the entire network layer from wireshark captures, because compression should not detect significant differences in non-MPC data. An example of this is packet retransmission, which do not add MPC information but increases capture size and introduces differences between captures.

In our assessment, of all Kolmogorov approaches, NCD is the most widely deployed solution, but some changes are introduced, such as Interleaving, to support large files. This problem focus on compressor limitations, such, lzip uses fixed-size dictionaries, which creates a problem that, when the dictionary is full, is redefined to an empty data set again.

The impact of Kolmogorov Complexity is focused usually on the use of NCD. From our opinion, this correlates with the availability of the libraries that implements this formula, contrarily to LZJD that was proposed/implemented recently.

## Figures and Tables

**Table 1:** Malware comparison table.

			Malware Dataset					
Articles	Type	Kolmogorov	Microsoft	Drebin	Arbor	Private	VirusWatch	Genome
[34]	Traces	NCD	○	○	●	●	○	○
[35]	Binary	NCD	○	○	○	●	●	○
[9]	Binary	NCD	●	○	○	○	○	●
[12,36]	Binary	LZJD	●	●	○	○	○	○

● = Use the dataset; ○ = Not using the dataset.
